# Parallel Evolution of Auditory Genes for Echolocation in Bats and Toothed Whales

**DOI:** 10.1371/journal.pgen.1002788

**Published:** 2012-06-28

**Authors:** Yong-Yi Shen, Lu Liang, Gui-Sheng Li, Robert W. Murphy, Ya-Ping Zhang

**Affiliations:** 1 State Key Laboratory of Genetic Resources and Evolution, and Yunnan Laboratory of Molecular Biology of Domestic Animals, Kunming Institute of Zoology, Chinese Academy of Sciences, Kunming, China; 2 Graduate School of the Chinese Academy of Sciences, Beijing, China; 3 Centre for Biodiversity and Conservation Biology, Royal Ontario Museum, Toronto, Ontario, Canada; 4 Laboratory for Conservation and Utilization of Bioresources, Yunnan University, Kunming, China; University of Michigan, United States of America

## Abstract

The ability of bats and toothed whales to echolocate is a remarkable case of convergent evolution. Previous genetic studies have documented parallel evolution of nucleotide sequences in *Prestin* and *KCNQ4*, both of which are associated with voltage motility during the cochlear amplification of signals. Echolocation involves complex mechanisms. The most important factors include cochlear amplification, nerve transmission, and signal re-coding. Herein, we screen three genes that play different roles in this auditory system. Cadherin 23 (*Cdh23*) and its ligand, protocadherin 15 (*Pcdh15*), are essential for bundling motility in the sensory hair. Otoferlin (*Otof*) responds to nerve signal transmission in the auditory inner hair cell. Signals of parallel evolution occur in all three genes in the three groups of echolocators—two groups of bats (Yangochiroptera and Rhinolophoidea) plus the dolphin. Significant signals of positive selection also occur in *Cdh23* in the Rhinolophoidea and dolphin, and *Pcdh15* in Yangochiroptera. In addition, adult echolocating bats have higher levels of *Otof* expression in the auditory cortex than do their embryos and non-echolocation bats. *Cdh23* and *Pcdh15* encode the upper and lower parts of tip-links, and both genes show signals of convergent evolution and positive selection in echolocators, implying that they may co-evolve to optimize cochlear amplification. Convergent evolution and expression patterns of *Otof* suggest the potential role of nerve and brain in echolocation. Our synthesis of gene sequence and gene expression analyses reveals that positive selection, parallel evolution, and perhaps co-evolution and gene expression affect multiple hearing genes that play different roles in audition, including voltage and bundle motility in cochlear amplification, nerve transmission, and brain function.

## Author Summary

The convergent origins of laryngeal echolocation in two groups of bats (Yangochiroptera and Rhinolophoidea) and toothed whales have long been a focus of interest for biologists. We screened three candidate genes—*Cdh23*, *Pcdh15*, and *Otof*—involved in different steps in the echolocation system. Signals of parallel evolution occurred in all three genes in the three groups of echolocators. *Cdh23* and *Pcdh15* constitute part of the mechanical link within the hair bundle of the ear. Both genes showed signals of both convergent evolution and positive selection, which implied they may have co-evolved to optimize cochlear amplification. Further, three lines of evidence suggest that *Otof* plays an important role in the transmission of signals in the brain during echolocation. First, the gene is more highly expressed in the auditory cortex of the brain in echolocating adult bats than in other cerebral cortexes. Second, this expression is higher in adult echolocating female bats than in their embryos, which do not use echolocation. Third, echolocators also have a higher level of expression in their cerebral cortexes than do non-echolocating bats. Taken with other evidence, the independent origins of echolocation involve the same genes that have evolved in precisely identical ways.

## Introduction

The ability of echolocation using ultrahigh frequency sounds occurs in two groups of bats (Yangochiroptera and Rhinolophoidea) and in toothed whales including dolphins [1]–[3]. These mammals use this complex bio-sonar system to assist with orientation and feeding [4], [5]. Echolocation by bats and dolphins provides an iconic example of either parallel or convergent evolution via natural selection.

Previous molecular studies on echolocation have mainly focused on the Organ of Corti. In this organ, the motor protein prestin plays a key role in voltage motility [6]–[8]. It appears to have undergone sequence convergence between bats and dolphins [1], [2], as well as within laryngeal echolocating bats [9]. Further, the voltage-gated potassium channel gene *KCNQ4* underwent parallel evolution in echolocating bats [3], [10]. Mammalian audition requires not only voltage motility, but also hair bundle motility, which is executed by outer hair cells in the cochlea [11]. Proteins encoded by the genes *Cdh23* and *Pcdh15* are essential to hair bundle motility [12]–[14], and their malfunctions in humans cause deafness in newborns and progressive retinitis pigmentosa (Usher syndrome type I) [15]. Homodimers of *Cdh23* and *Pcdh15* directly link to each other via their amino termini; they form the upper and lower part of tip-links, respectively (Figure S1), which lie between the stereocilia within the hair bundle [14], [16], [17]. The auditory system involves the perception and enhancement of sound signals, as well as transformation of the mechanical signals to ion fluxes in inner hair cells. Management of the electric signals to the brain involves a series of nerve channel openings [18]. Genetic mutations in the gene encoding otoferlin (*Otof*) cause a clinical, autosomal recessive nonsyndromic form of prelingual and sensorineural deafness [19]–[21]. This protein that may act as the major Ca^2+^ sensor that triggers membrane fusion at the ribbon synapse of the auditory inner hair cell [22]. Although the above functions are involved in the conversion of sound signals into electrical impulses in the inner ear, the expression of *Otof* also occurs in neurons and nerve fibers in the brain [23].

The molecular mechanism of voltage motility in echolocation is widely studied. Echolocation is a complex system that includes signal reception by hair cells in the Organ of Corti, nerve transmission, and signal processing in the brain [24]. Therefore, herein we investigate the gene sequence evolution of *Cdh23*, *Pcdh15*, and *Otof*. These proteins function in different steps during echolocation. Because the brain modulates sensory information from peripheral sensory organs [25], and because *Otof* is involved in transferring sound signal by electrical impulses, we also examine the expression patterns of *Otof* in the cerebral cortexes of different species. We synthesize evidence from sequences and expressions to study the convergent evolution of echolocation in bats and dolphins.

## Results

### Tree analyses

**Figure 1:**
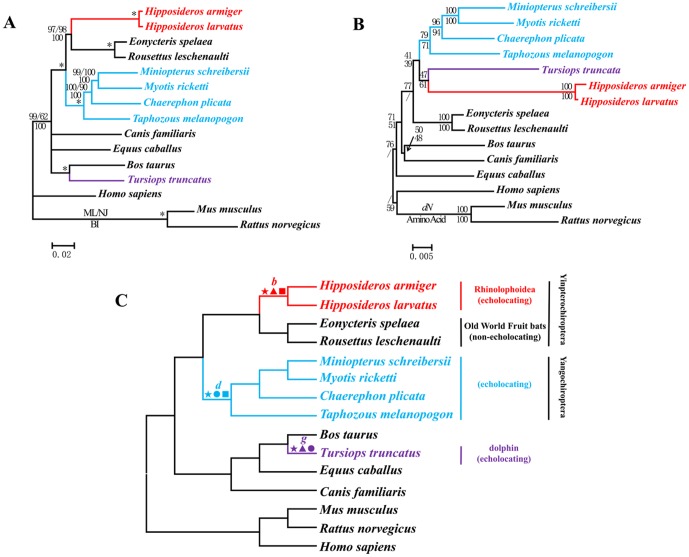
Parallel evolution of *Cdh23* in bats and dolphins. (A) Gene tree of *Cdh23* based on nucleotide sequences that is consistent with the species tree. Numbers above the branches are the ML and NJ bootstrap values, respectively. Numbers below the branches are Bayesian posterior probabilities. * indicates all values equal 100. (B) Gene tree of *Cdh23* based on nonsynonymous mutations and the amino acid sequences. Numbers along the branches are NJ bootstrap values. (C) Species tree based on previous studies [26]–[28]. Symbols above the branches correspond to amino acid replacements. ★ indicates a parallel amino acid replacement presenting on branches *b*, *d*, and *g*: R204Q. ▴ indicates parallel amino acid replacements presenting on branches *b* and *g*: R204Q, D517N, P518A, S639N, N737S, S747T, A1080S, K1141T, S1314T, A1382S, I1673V, N1697D, L1960F, L1974I, A2146V, G2229S, V2427I, T2439R, R2639K, Q2725L, and N3180S. • indicates a parallel amino acid replacement presenting on branches *d* and *g*: R204Q. ▪ indicates parallel amino acid replacements presenting on branches *b* and *d*: R204Q, R535K, T904I, and V1691I.

We built ML, BI, and NJ trees based on both nucleotide and amino acid sequences. The length of aligned nucleotides for *Cdh23* was 9657 base pairs (bp). The gene trees for *Cdh23* based on nucleotide sequences (Figure 1A) were basically the same as the well-accepted species tree (Figure 1C) [26]–[28] in all methods of tree-building. Echolocating *Hipposideros* clustered with Old World (OW) fruit bats, which represented the Yinpterochiroptera. The genera *Taphozous*, *Chaerephon*, *Miniopterus*, and *Myotis* clustered together, forming the Yangochiroptera, the sister group of Yinpterochiroptera. However, the topologies based on amino acid sequences (Figure 1B) differed substantially from those based on nucleotide sequences. All echolocators incorrectly grouped together and then became the sister-group of the OW fruit bats, which do not possess the ability of laryngeal echolocation. The topology of the tree based on synonymous sites of *Cdh23* was nearly the same as that based on nucleotide sequences, as well as the species-tree. The NJ tree using nonsynonymous changes was congruent with the amino acid tree (Figure 1B).

**Figure 2:**
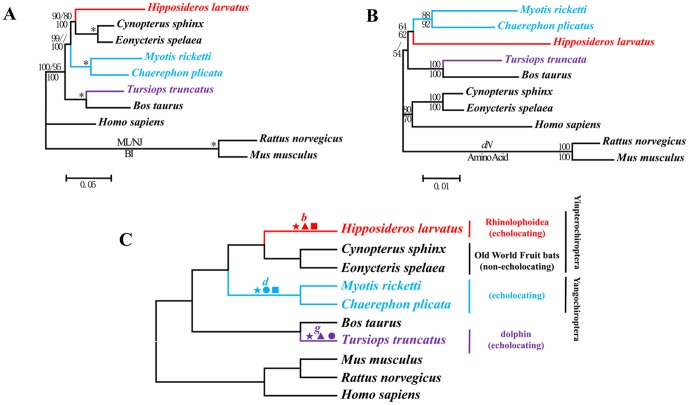
Parallel evolution of *Pcdh15* in bats and dolphins. (A) Gene tree of *Pcdh15* based on nucleotide sequences. Numbers above the branches are the ML and NJ bootstrap values, respectively. Numbers below the branches are Bayesian posterior probabilities. * indicates all values equal 100. (B) Gene tree of *Pcdh15* based on nonsynonymous changes and amino acid sequences. Numbers along the branches are NJ bootstrap values. (C) Species tree based on the previous studies [26]–[28]. Symbols above the branches correspond to amino acid replacements. ★ indicates parallel amino acid replacements presenting on branches *b*, *d*, and *g*: M946I and D1278E. ▴ indicates parallel amino acid replacements presenting on branches *b* and *g*: N218D, Q310E, E393V, T427S, A433V, I438V, T490I, V546F, I643V, N666K, K820R, I853V, K856T, M946I, V952A, R999L, T1139R, F1160L, A1173S, K1275R, D1278E, and I1404V. • indicates parallel amino acid replacements presenting on branches *d* and *g*: A726D, M946I, and D1278E. ▪ indicates parallel amino acid replacements presenting on branches *b* and *d*: L423V, Q468P, H765Y, F876L, M946I, F984S, V1019I, and D1278E.

For *Pcdh15*, the aligned length was 5835 bp. The tree based on its nucleotide sequences (Figure 2A) depicted the well-accepted species tree (Figure 2C) and the nodes received high bootstrap values. In contrast, the amino acid trees (Figure 2B) clustered all echolocating bats together, and this arrangement differed from the nucleotide trees. As with *Cdh23*, the topology of the tree based on synonymous sites was virtually identical to that based on nucleotide sequences, while the tree based on the nonsynonymous changes was congruent with the amino acid tree (Figure 2B).

**Figure 3:**
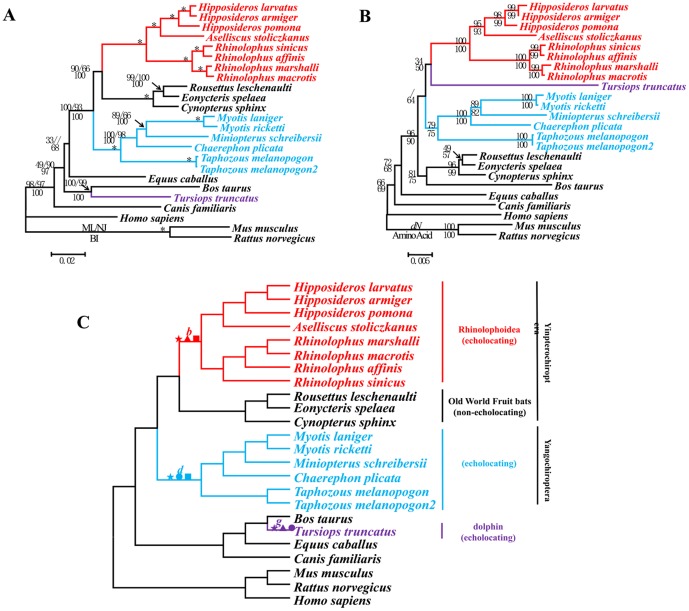
Parallel evolution of *Otof* in bats and dolphins. (A) Gene tree of *Otof* based on nucleotide sequences. Numbers above the branches are the ML and NJ bootstrap values, respectively. Numbers below the branches are Bayesian posterior probabilities. * indicates all values equal 100. (B) Gene tree of *Otof* based on the nonsynonymous changes and corresponding amino acid sequences. Numbers on the branches are NJ bootstrap values. (C) Species tree based on the previous studies [26]–[28]. Symbols above the branches correspond to amino acid replacements. ★ indicates a parallel amino acid replacement presenting on branches *b*, *d*, and *g*: D396E. ▴ indicates parallel amino acid replacements presenting on branches *b* and *g*: P191H, G213A, D396E, and V440M. • indicates parallel amino acid replacements presenting on branches *d* and *g*: D396E and R1238H. ▪ indicates a parallel amino acid replacement presenting on branches *b* and *d*: D396E.

The sequenced coding region of *Otof* varied from 5363 to 5645 bp. As common in the Yinpterochiroptera, a 20 bp deletion occurred from bp site 1248 to 1267. The trees for *Otof* showed patterns similar to those of the previous two genes; the amino acid trees and NJ topology for nonsynonymous sites incorrectly clustered all echolocators (Figure 3B), and this association conflicted with the nucleotide trees (Figure 3A). Again, the nucleotide trees were consistent with the species tree (Figure 3C).

### Analyses of convergent/parallel evolution

Branches *b*, *d*, and *g* in the species tree (Figure 1C, Figure 2C, Figure 3C) lead to mammals that have the ability to echolocate. Branch *b* represented the common ancestor of the Rhinolophoidea, *d* of the Yangochiroptera, and *g* of the dolphin. We reconstructed ancestral nodes and mapped amino acid changes along these branches. For *Cdh23*, branches *b*, *d*, and *g* shared one amino acid change (R204Q). Branches *b* and *g* shared the following 21 parallel mutations: R204Q, D517N, P518A, S639N, N737S, S747T, A1080S, K1141T, S1314T, A1382S, I1673V, N1697D, L1960F, L1974I, A2146V, G2229S, V2427I, T2439R, R2639K, Q2725L, and N3180S. Parallel evolution of these two branches statistically differed from random expectations (*P*<0.001). Branches *b* and *d* had four parallel mutations (R204Q, R535K, T904I, and V1691I). Again, parallel evolution was statistically significant (*P*<0.001). All changed sites were mapped in Figure 1C. The positions of these sites in the domain structure of *Cdh23* were mapped in Figure S2. We constructed a BI tree from the aligned amino acids of *Cdh23* while excluding all parallel-evolved sites. The BI tree agreed with the species tree (Figure S3).

Two parallel mutations in *Pcdh15* (I946M and E1278D) were shared by branches *b*, *d*, and *g*. Parallel evolution was statistically significant (*P*<0.001) between branches *b* and *g* for the following 22 parallel mutations: N218D, Q310E, E393V, T427S, A433V, I438V, T490I, V546F, I643V, N666K, K820R, I853V, K856T, M946I, V952A, R999L, T1139R, F1160L, A1173S, K1275R, D1278E, and I1404V. Parallel evolution was also statistically significant (*P*<0.001) between branches *d* and *g* for three parallel mutations: A726D, M946I, and D1278E. Finally, eight parallel mutations occurred between branches *b* and *d*, including L423V, Q468P, H765Y, F876L, M946I, F984S, V1019I, and D1278E (Figure 2C). Parallel evolution between these two branches was statistically significant (*P*<0.001). The positions of these sites in the domain structure of *Pcdh15* were mapped in Figure S4. The BI tree based on amino acids excluding all parallel-evolved sites differed somewhat with the species tree, but it did not group echolocators together (Figure S5).

*Otof* had one amino acid change shared among echolocators (D396E). The four parallel-evolved sites along branches *b* and *g* were P191H, G213A, D396E, and V440M (Figure 3C), and parallel evolution was statistically significant (*P*<0.001) between these two branches. Parallel evolution of branch *d* and *g* was also significant (*P*<0.001). The conserved sites were shown in Figure S6. The BI tree based on amino acids while excluding all parallel-evolved sites agreed with the species tree (Figure S7).

### Selective pressure analysis

Selective pressure was evaluated using the PAML package and test results were presented in Table 1 and Table S1. The one-ratio model obtained an average ω (*Ka/Ks* ratio) of 0.0546 (lnL = −35796.7768) for *Cdh23*. For specific branches, we alternatively set the echolocating bats (Rhinolophoidea and Yangochiroptera; branches *b* and *d* in Figure 1C, respectively) or dolphin (branch *g* in Figure 1C) as foreground branch. In both conditions, although the ratios of ω for the foreground branches were greater than background branches, they were less than 1 (ω_branch (*b+d*)_ = 0.1107 while ω_0_ = 0.0500; ω_branch *g*_ = 0.1145 while ω_0_ = 0.0516). When considering all echolocators together, ω_echolocators_ was 0.1126 for the foreground branch compared with 0.0465 for background branch (2*Δ*l = 660.6044, df = 1, *P*<0.001). For the branch-site models, test 2 (Model A vs. null model) was used to control the false positive signals. Branch *b* was detected to have undergone significant positive selection (*P*<0.01), and eight sites with BEB values >0.90 (213 A 0.984, 692 F 0.984, 1165 N 0.989, 1171 S 0.958, 1256 D 0.989, 1356 I 0.990, 1687 T 0.925, and 2492 L 0.970). When we set the dolphin as the foreground branch (branch *g*), significantly positive selection was also detected (2*Δ*l = 10.7224, df = 1, *P*<0.01), and six sites (580 S 0.974, 840 H 0.973, 1011 H 0.973, 1014 T 0.978, 2133 T 0.952, and 2224 T 0.974) with BEB values >0.90. When all echolocating species were combined as the foreground branch, again significant positive selection signals were obtained (2*Δ*l = 8.6179, df = 1, *P*<0.01). The positions of these positively selected sites were mapped in Figure S2.

**Table 1:** Summary of selective pressure analysis for the hearing genes *Cdh23*, *Pcdh15*, and *Otof*.

Gene	branch	Parameter Estimated					2*Δ*lnL*P* value	Positively Selected Sites (BEB Analysis)
		**site class**	**0**	**1**	**2a**	**2b**		
*Cdh23*	branch *b*	proportion	0.92097	0.05178	0.02580	0.00145	**10.4158** **P<0.01**	**213 A**, **692 F**, **1165 N**, **1171 S**, **1256 D**, **1356 I**, **2492 L**
		background ω	0.03156	1.00000	0.03156	1.00000		
		foreground ω	0.03156	1.00000	3.85446	3.85446		
	branch *d*	proportion	0.94313	0.05687	0.00000	0.00000	0	
		background ω	0.03347	1.00000	0.03347	1.00000		
		foreground ω	0.03347	1.00000	1.00000	1.00000		
	branch *g*	proportion	0.92922	0.05575	0.01418	0.00085	**10.7224** **P<0.01**	**580 S**, **840 H**, **1011 H**, **1014 T**, **2133 T**, **2224 T**
		background ω	0.03168	1.00000	0.03168	1.00000		
		foreground ω	0.03168	1.00000	5.94936	5.94936		
*Pcdh15*	Branch *b*	proportion	0.79672	0.11161	0.08040	0.01126	2.6591P = 0.1	**454 H**, **816 T**
		background ω	0.02983	1.00000	0.02983	1.00000		
		foreground ω	0.02983	1.00000	2.00038	2.00038		
	branch *d*	proportion	0.86720	0.12926	0.00308	0.00046	**6.4259** **P<0.05**	573 T 0.921
		background ω	0.03721	1.00000	0.03721	1.00000		
		foreground ω	0.03721	1.00000	24.77802	24.77802		
	Branch *g*	proportion	0.81368	0.11630	0.06127	0.00876	2.9014P = 0.09	**1584D**
		background ω	0.03403	1.00000	0.03403	1.00000		
		foreground ω	0.03403	1.00000	2.72477	2.72477		
*Otof*	Branch *b*	proportion	0.95114	0.03918	0.00930	0.00038	0.7680P<0.5	
		background ω	0.02313	1.00000	0.02313	1.00000		
		foreground ω	0.02313	1.00000	3.10544	3.10544		
	branch *d*	proportion	0.95968	0.04032	0.00000	0.00000	0P = 1	
		background ω	0.02352	1.00000	0.02352	1.00000		
		foreground ω	0.02352	1.00000	1.00000	1.00000		
	Branch *g*	proportion	0.92038	0.03758	0.04039	0.00165	0P = 1	**944 L**
		background ω	0.02221	1.00000	0.02221	1.00000		
		foreground ω	0.02221	1.00000	1.00000	1.00000		

For *Pcdh15*, we implemented the same series of analysis as for *Cdh23*. All branch models were significantly better than the null model that fixed ω of the foreground branch to 1, although the value of ω never exceeded 1 (ω_branch (*b+d*)_ = 0.3211, ω_0_ = 0.0998, 2*Δ*l = 83.0381, df = 1, *P*<0.001; ω_branch *g*_ = 0.4469, ω_0_ = 0.1109, 2*Δ*l = 16.6620, df = 1, *P*<0.001; and ω_echolocators_ = 0.3561, ω_0_ = 0.0851, 2*Δ*l = 96.3532, df = 1, *P*<0.001). For the branch-site models, a significant signal of positive selection was detected on branch *d* and one site had a BEB value >0.90 (*P*<0.05). The position of this positively selected site was mapped in Figure S4.

For *Otof*, the ω ratios in branch models were greater than ratios of the background branches, but less than 1 (ω_branch (*b+d*)_ = 0.0572, ω_0_ = 0.0364; ω_branch *g*_ = 0.0886, ω_0_ = 0.0352; and ω_echolocators_ = 0.0732 with ω_0_ = 0.0340). Branch-site models did not detect any signals of positive selection in the echolocators (Table 1).

### Gene expression pattern analysis

**Figure 4:**
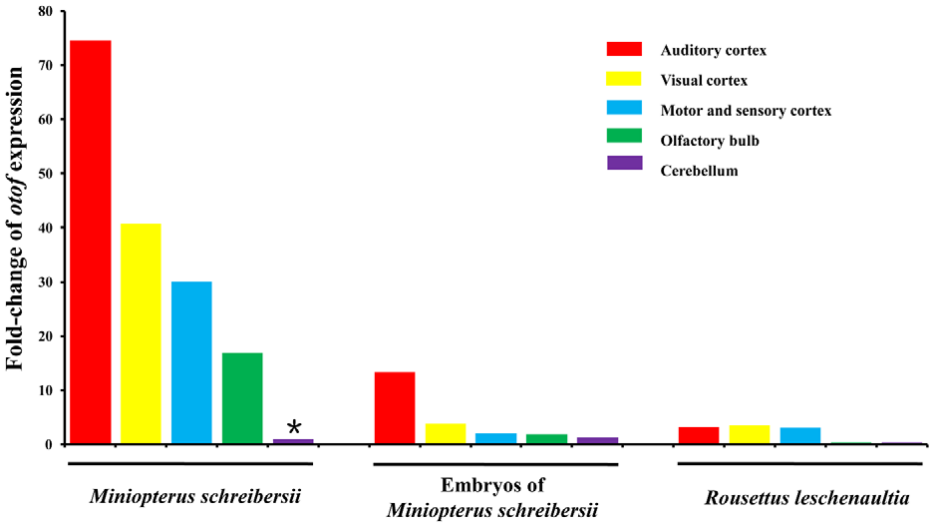
Expression patterns of the gene *Otof* in different cerebral cortexes of bats. The * indicates baseline value.

Real-Time PCR was used to assess expression patterns of *Otof* in the brain. We set the mean value of gene expression in the cerebellum of the adult Common Bent-wing Bat (*Miniopterus schreibersii*) as the baseline unit (marked * in Figure 4), and then compared expression patterns of *Otof* in different cortexes of the brain (Figure 4 and Table S2). The level of expression in the auditory cortex was more than 70-fold that of the baseline value. The levels of expression from the visual cortex, and motor and sensory cortex were more than 40-fold and 30-fold greater than the baseline value, respectively, whereas the expression in the olfactory bulb was nearly 17-fold greater. We compared this expression pattern with that of embryos. Expression levels of *Otof* in the auditory cortexes of three embryonic Common Bent-wing Bats were nearly 13-fold the baseline value and the visual cortex was over 3-fold. In contrast, expression levels in motor and sensory cortex, olfactory bulb, and cerebellum were similar to the baseline values. In adult Old World Fruit Bats (*Rousettus leschenaultii*), which do not echolocate, expression levels in the auditory cortex, visual cortex, and motor and sensory cortex were all around 3-fold greater than the baseline value, and expression levels in the olfactory bulb and cerebellum were less than the baseline value.

## Discussion

Morphological development is very complex and involves a suite of genes. Some mammals have independently developed similar features, such as the ability to echolocate objects. Echolocation by bats and dolphins provides an extreme example of parallel or convergent evolution. Although the morphologies of sending and receiving sonic signals differ greatly, the hearing of ultrasonic sounds and the mechanisms of decoding signals are shared [29], [30]. Thus, the genes coding for the auditory system become ideal candidates for adaptive evolution at the molecular level during echolocation.

The genes *Cdh23* and *Pcdh15* are essential to hair bundle motility [12]–[14], [31], [32]. *Otof* encodes a protein that may act as the major Ca^2+^ sensor to trigger membrane fusion at the auditory inner hair cell ribbon synapse [22]. The three genes are involved in different steps of the hearing system. Their nucleotide gene trees are largely congruent with the species tree and the nodes enjoy high support. In contrast, the amino acid trees conflict with the species tree; all unite the echolocators. Further, trees based on nonsynonymous sites have topologies similar to those based on corresponding amino acids, and yet the trees based on synonymous mutations do not show this pattern. Clearly, the difference in branching order is the result of amino acid changes (nonsynonymous mutations).

Three independent lineages of mammals can echolocate and it is important to identify whether this involves convergent or parallel evolution. If echolocation involves convergent evolution, then similar traits or functions independently emerge in two or more lineages from different ancestral states. Parallel evolution differs in that similar ancestral traits descend into similar extant states in different lineages [33].

Reconstructions of the ancestral sequences of the internal nodes can detect which amino acid changes cause the incongruence between the amino acid and species trees. A suite of sites show parallel changes in these echolocators, and these changes have a statistically significant signal of parallel evolution. Twenty-one amino acid sites converge on the same residue in branch *g* and branch *b* and with a highly significant probability (*P*<0.001). One convergent site appears in branches *g* and *d* (*P*<0.001), and four parallel sites occur in branches *b* and *d* (*P*<0.001) in *Cdh23*. In *Pcdh15*, 22 and three convergent sites occur between branch *g* and branches *b* and *d*, respectively (*P*<0.001 in both cases) and eight parallel sites have been identified between branches *b* and *d* (*P*<0.001). Four parallel sites in *Otof* occur on branches *g* and *b* (*P*<0.001). Convergent evolution is not accommodated by current phylogenetic methods and it can strongly mislead phylogenetic inference [34]. Upon excluding the parallel-evolved amino acid sites, the reconstructed amino acid trees did not incorrectly unite all echolocators (Figures S3, S5, S7). Thus, the discovery of multiple parallel-evolving amino acid sites explains the unnatural uniting of echolocators in the amino acid trees.

In addition to the parallel evolution of *Prestin* [1], [2], [9] and *KCNQ4* [3], our analyses document a high level of complexity in a large-scale, multigene adaptation. Functional assays have proven that parallel and convergent amino acid changes are responsible for parallel and convergent functional changes [35], [36]. Thus, parallel evolution of gene sequences may have driven phenotypic and functional convergence in echolocating bats and dolphins. Further functional assays are needed to affirm this association.

Our analyses of selection pressure detect signals of positive selection in *Cdh23* on the branches leading to the Rhinolophoidea and dolphin. The same occurs in *Pcdh15* along the branch leading to Yinpterochiroptera (see Table 1 and Table S1). Positive selection appears to have acted on these genes to fit the requirements for echolocation. Whereas outer hair cells amplify sound by somatic [37], [38] or ciliary [39], [40] mechanisms, inner hair cells are passive detectors of the amplified vibratory signal. The signals are converted into electrical impulses by activating fibers of the cochlear (auditory) nerve [41], which then sends the signals to higher auditory processing centers in the brain. *Prestin* mediates the voltage somatic motility unique to mammals [42]–[44]. Part of the cochlear amplification, *Cdh23* and *Pcdh15* participate in hair bundle motility [39], [45], [46]. *Otof* participates by releasing neurotransmitter to nerves [22]. Convergent evolution and positive selection on these genes reflect the pathway from receipt of signal to signal amplification, and then to neural transduction. All parts of the pathway are involved in the auditory system, and thus may play important roles in echolocation.

Homodimers of *Cdh23* and *Pcdh15* directly link to each other via their amino termini, and they constitute the upper and lower part of tip-links, respectively, that lie between the stereocilia within the hair bundle [14], [16], [17]. Sequence alignments suggest that *Cdh23* and *Pcdh15* have 27 and 11 extracellular cadherin repeats, respectively [47]. Functional evidence encompassing a classical genetic approach shows that mutations at these two cadherin proteins can interact to cause hearing loss in digenic heterozygotes of both mice and humans [48]. *Cdh23* and *Pcdh15* appear to have undergone both convergent functional evolution and positive selection in echolocators. This finding suggests a strong interaction between the proteins and co-evolution is likely to have optimized their function in cochlear amplification for the development of echolocation.

Dolphins and bats employ different tools to echolocate. Whereas the larynx generates sound in echolocating bats [29], the monkey lip/dorsal bursa complex does the same in dolphins [30]. In bats, the sound involves a constant frequency (CF) and frequency modulation (FM), but dolphins use FM and amplitude modulation (AM) [49]. Convergence occurs via both auditory systems being adapted to receiving and processing ultrahigh frequency sounds. Our study discovers evidence of parallel sequence evolution in three genes involved in hearing and this may indicate a genetic basis for echolocation.

Sequence evolution only tells a part of the story. Expression pattern is usually a strong indicator of protein-demand and function [50], [51]. The central auditory system plays the crucial role of receiving impulses from auditory nerves and sending messages back to the cochlea [52]. Because *Otof* plays a role in neural signal transmission, we have evaluated its patterns of expression in different cerebral cortexes. *Otof* is most highly expressed in the auditory cortex of echolocating adult female Common Bent-Wing Bats. Adult females, which frequently use ultrasonic sounds to explore their environments, have higher levels of expression than their embryos, which do not use echolocation. Further, a comparison of expression of *Otof* between the brains of an adult echolocating bat (Common Bent-Wing Bat, *Miniopterus schreibersii*) and a non-echolocating bat (Old World Fruit Bat, *Rousettus leschenaultii*) reveals a higher level of expression in the former. Indeed, the gene exhibits great differences in levels of expression in both different cerebral cortexes and in species with or without the ability to echolocate.

Echolocation signals begin at the hair cells in the Organ of Corti, continue along the auditory nerve, and terminate in the auditory cortex of the brain [24]. Combined with sequence data and expression data, we conclude that multiple instances of parallel sequence evolution are involved in genes in different parts of auditory system between the three groups of echolocators. This occurs not only in well-studied voltage motility, but also bundle motility, and not only in cochlear amplification, but also in neural transduction. Further, co-evolution optimizes the function of homodimers of *Cdh23* and *Pcdh15* in cochlear amplification. The expression pattern of *Otof* in different cerebral cortexes implies that the evolution of gene expression might be required for echolocation. In conclusion, we synthesize gene sequence and gene expression analyses and conclude that positive selection, convergent evolution, and perhaps co-evolution and gene expression evolution play roles in audition (voltage motility and bundle motility in cochlear amplification, nerve transmission, and brain) during the independent origins of echolocation in bats and dolphins.

## Materials and Methods

### Ethics statement

All research involving animals used in this study followed the guidelines and bylaws on animal experimentation. Bats were anesthetized using an intraperitoneal injection of sodium pentobarbital (C_11_H_17_N_2_NaO_3_) at a dosage of 100 mg per kg body weight. Following anesthesia, bats were euthanized and then their brains were sampled. Protocols were approved by the Ethics and Experimental Animal Committee of the Kunming Institute of Zoology, Chinese Academy of Sciences.

### Source of data and primary treatments

Sixteen species of bats were used in our analysis (Table S3). Total RNA was isolated from the brain using a RNAiso Plus Kit (Takara, China), and RT-PCR was performed on 2 ug of RNA using the PrimeScript RT-PCR Kit (Takara, China) to obtain cDNA. Subsequently, genes were amplified from cDNA using gene-specific primers (Table S4). PCR products were purified on a 1% agarose gel and a Watson Gel Purification Kit (Watson BioTechnology, Shanghai), and finally transformed into the pMD18-T vector (Takara, China). Each strand was sequenced in both directions with an ABI 3730 sequencer. RNA samples of brain were not available for the dolphin (*Tursiops truncatus*), so genomic sequences were amplified from total genomic DNA, which was extracted from muscle tissue using a standard 3-step phenol/chloroform extraction method [53].

Raw nucleotide sequences were edited using Lasergene SeqMan software (DNASTAR Inc., Madison, WI, USA). Newly determined sequences were deposited in GenBank (Accession numbers JF808081–JF808094, JQ284400–JQ284430). The sequences of background species came from the Ensembl database (Release 66) and those of high quality were used (Table S3). All the sequences were aligned using ClustalX 1.81 [54] and then visually checked for accuracy (the aligned sequences are available by request).

### Phylogenetic and molecular evolutionary analyses

The best-fit models were selected by jModeltest v0.1.1 [55], [56] for nucleotide sequences and ProtTest 3.0 beta [57] for amino acid sequences. Maximum likelihood (ML) trees were reconstructed by PAUP* [58] with 1,000 replications, and Bayesian inference (BI) trees were reconstructed by MrBayes 3.1.2 with 1,000,000 replications [59], [60].

Neighbor-joining (NJ) phenograms were based on Kimura 2-parameter corrected distances for nucleotide sequences and uncorrected P-distances for amino acid sequences, each with 1,000 bootstrap replications. We implemented the Li-Wu-Luo method [61] to reconstruct NJ trees based on both synonymous and nonsynonymous sites.

The sequences of the internal nodes were reconstructed using distance-based Bayesian methods, which included the branch lengths estimated by the least squares method and the ancestral amino acids inferred by the Bayesian approach. These data were used to obtain an unbiased estimate of the true probability [62]. Convergent and parallel amino acid substitutions along each lineage were detected. The statistical significance of these amino acid changes was tested with the method developed by Zhang and Kumar [63].

The CODEML program in PAML 4 [64] was used to detect selective pressure. The species tree [26]–[28] was used as guide tree for analysis. Four models of evolution were used: one-ratio model, free-ratio model, branch models, and branch-site models. For the last two models, three groups of echolocators were set as the foreground branch to detect whether or not they had undergone positive selection.

### Expressional data analysis

We sampled auditory cortex, visual cortex, motor and sensory cortex, olfactory bulb, and cerebellum from euthanized adult and embryonic *Miniopterus schreibersii*, and adult *Rousettus leschenaultii*, which represented the adult echolocating bats, embryonic echolocating bats, and adult non-echolocating bats, respectively. Tissue was selected according the human brain atlas [65]. These tissues were stored in liquid nitrogen. Total RNA was isolated using a RNAiso Plus kit (Takara, China). Next, DNA-free RNA samples were condensed using a RNAqueous-4PCR Kit (Applied Biosystems, US). We synthesized cDNA using a PrimeScript RT-PCR Kit (Takara, China), and then used it in Real-Time PCR. TaqMan Gene Expression Assays were custom designed by Applied Biosystems based on our sequencing data of *Otof* and *Actb* in bats. The assay details were listed in Table S5. Sequences of *Otof* and *Actb* were amplified and detected using an ABI PRISM 7000 Sequence Detection System with a PCR profile as follows: 50°C for 2 min, 95°C for 10 min, followed by 40 cycles at 95°C for 15 s, and 60°C for 1 min. The products were purified and sequenced in both directions with an ABI 3730 sequencer to insure the assays' specificity. Real-Time PCR was performed on 96-well reaction plates in a 20 µl reaction volume containing 100 ng of cDNA per reaction with TaqMan Universal Master Mix II (Applied Biosystems, US). For each group, three individuals were used and each sample was performed three times. Expression data for the target gene *Otof* were normalized relative to the housekeeping gene *Actb*. Raw data were obtained and analyzed using the 7000 SDS 1.1 software (Applied Biosystems, US). The comparative C_T_ method (*ΔΔ*Ct) was chosen to further calculate the relative expressions between different groups.

## Supporting Information

Figure S1 Model of *Cdh23* and *Pcdh15* localization at tip-links [16].
(TIF)

Figure S2 Domain structure of *Cdh23* (SS, signal sequence; EC, ectodomain; HTM, transmembrane domain), and the positions of the positively selected sites and parallel-evolving sites.
(TIF)

Figure S3 The BI tree for *Cdh23* based on the amino acid sequences excluding all parallel-evolved sites.
(TIF)

Figure S4 Domain structure of *Pcdh15* (SS, signal sequence; EC, ectodomain; HTM, transmembrane domain), and the positions of the positively selected sites that excluded all parallel-evolving sites.
(TIF)

Figure S5 The BI tree for *Pcdh15* based on the amino acid sequences excluding all parallel-evolved sites.
(TIF)

Figure S6 Parallel-evolved sites of *Otof*.
(TIF)

Figure S7 The BI tree for *Otof* based on the amino acid sequences excluding all parallel-evolved sites.
(TIF)

Table S1 Details of the selective pressure analyses on the three hearing genes.
(DOCX)

Table S2 Expression levels of *Otof* in the Common Bent-wing Bat and Old World Fruit Bat.
(DOCX)

Table S3 Species and their accession numbers for the genes *Cdh23*, *Pcdh15*, *Otof*, and *Actb* used in this research.
(DOCX)

Table S4 The primers used for amplifying and sequencing the three hearing genes.
(DOCX)

Table S5 Details of the TaqMan gene expression assays.
(DOCX)
