# Supplementary figures and images for: Parallel Evolution of Auditory Genes for Echolocation in Bats and Toothed Whales
Source: PLoS Genet. 2012 Jun 28;8(6):e1002788. doi: 10.1371/journal.pgen.1002788 (PMC3386236; doi:10.1371/journal.pgen.1002788)

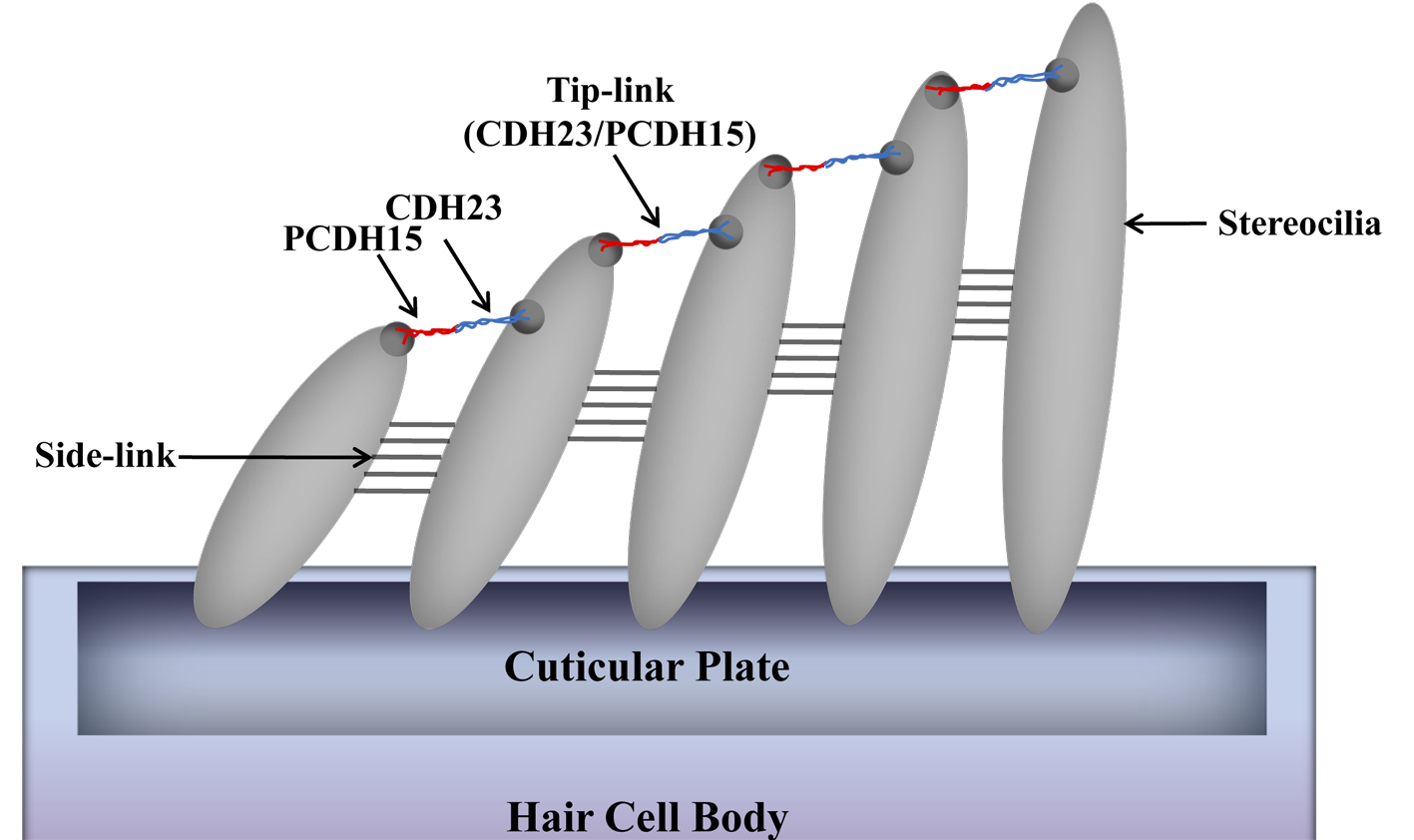

Supplement: Figure S1 — Model of Cdh23 and Pcdh15 localization at tip-links [16]. (TIF) [file pgen.1002788.s001.tif]

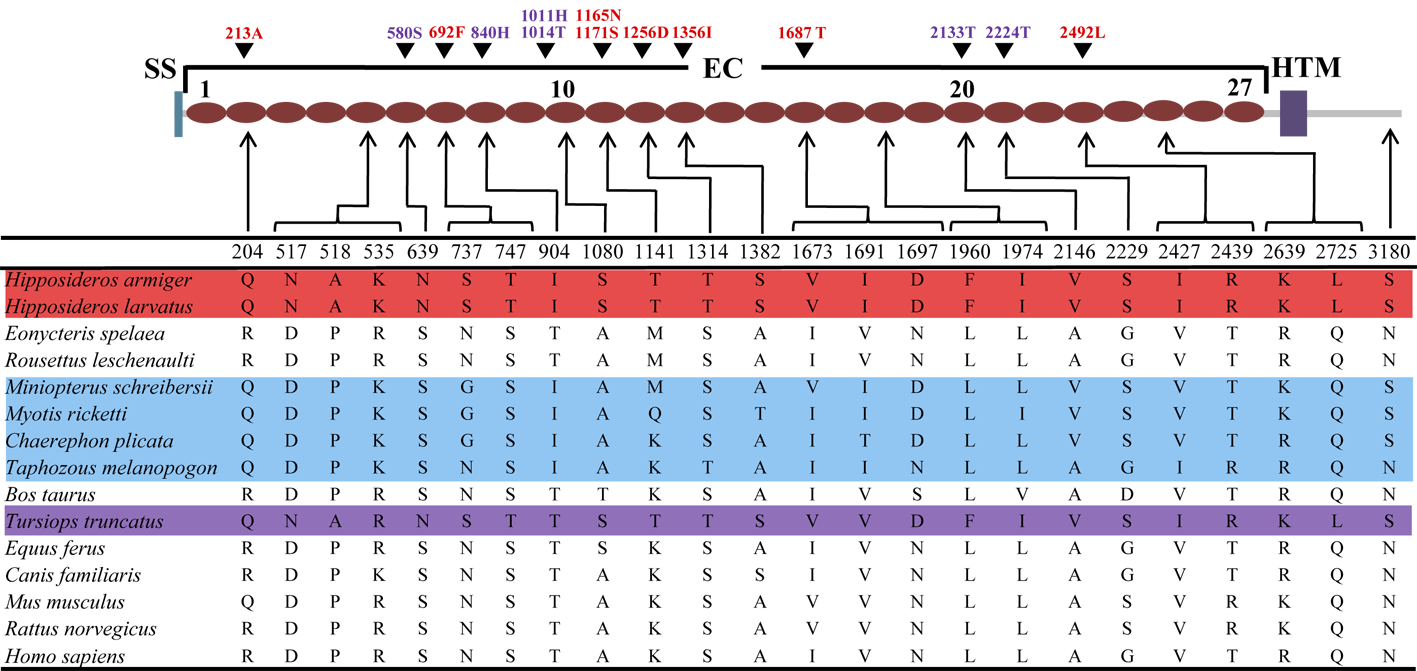

Supplement: Figure S2 — Domain structure of Cdh23 (SS, signal sequence; EC, ectodomain; HTM, transmembrane domain), and the positions of the positively selected sites and parallel-evolving sites. (TIF) [file pgen.1002788.s002.tif]

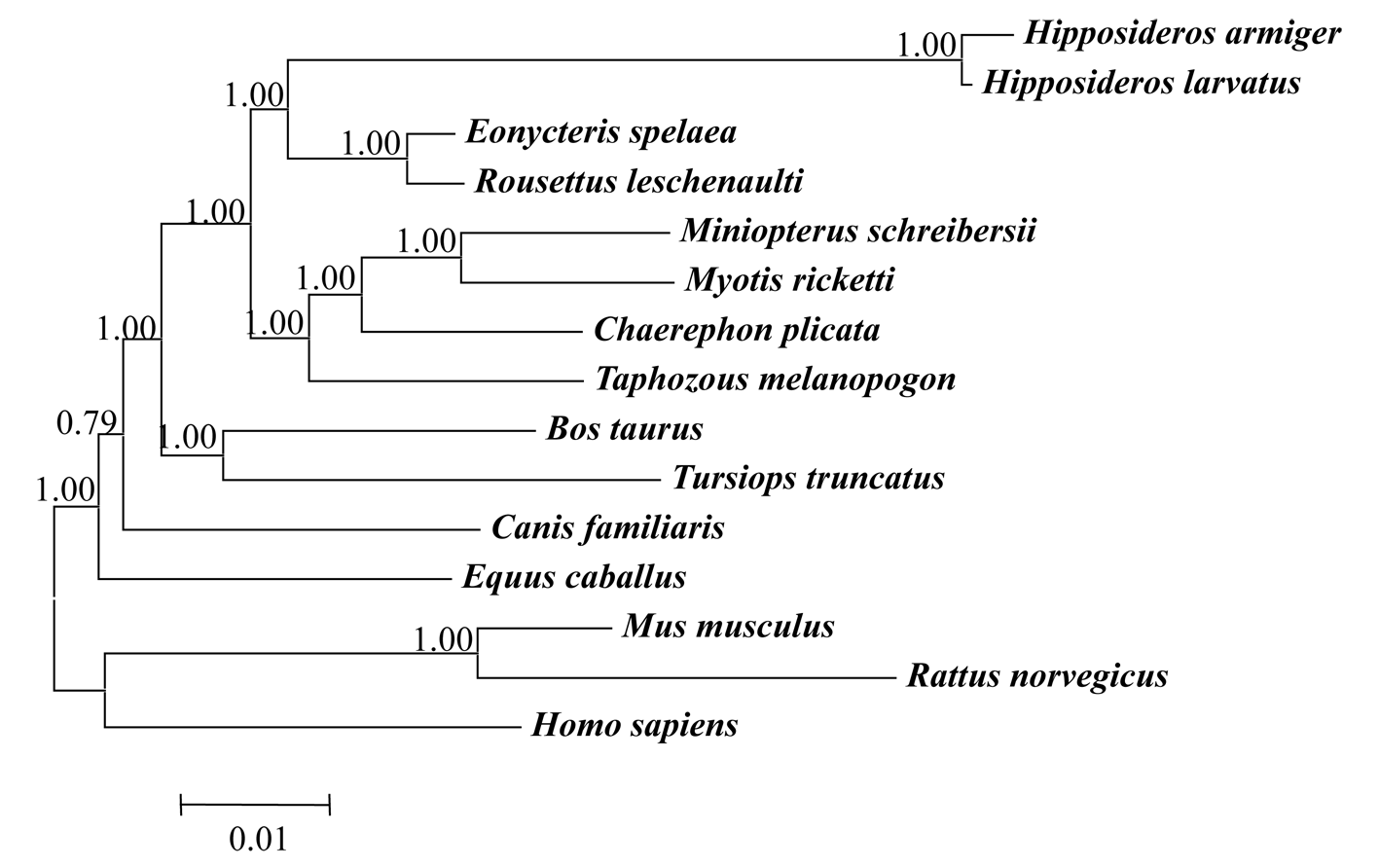

Supplement: Figure S3 — The BI tree for Cdh23 based on the amino acid sequences excluding all parallel-evolved sites. (TIF) [file pgen.1002788.s003.tif]

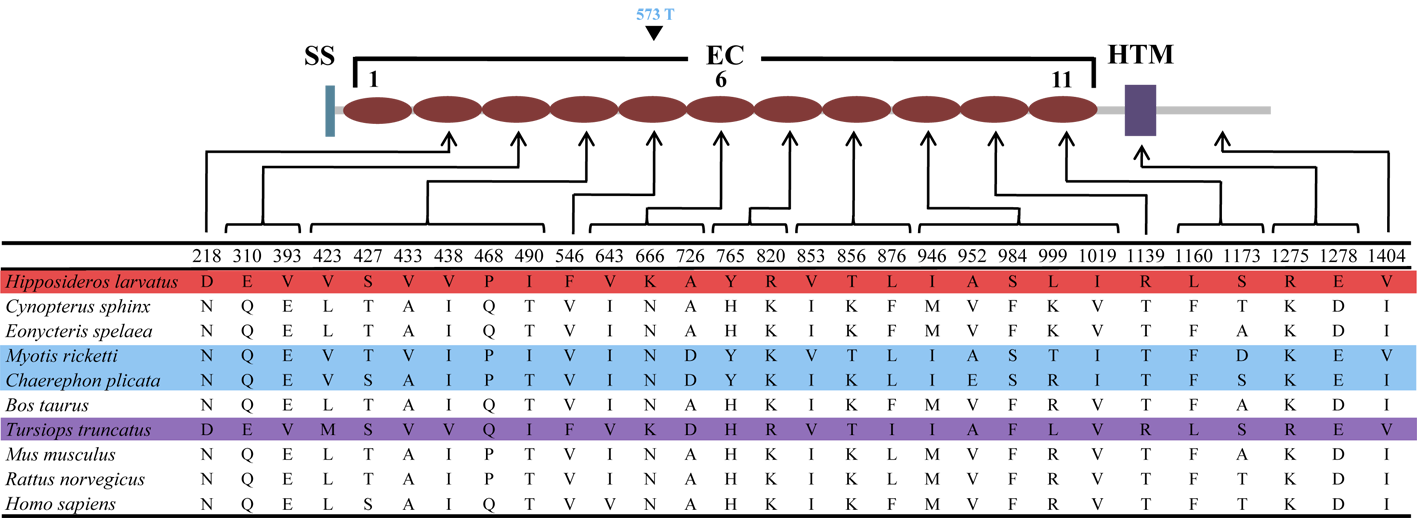

Supplement: Figure S4 — Domain structure of Pcdh15 (SS, signal sequence; EC, ectodomain; HTM, transmembrane domain), and the positions of the positively selected sites that excluded all parallel-evolving sites. (TIF) [file pgen.1002788.s004.tif]

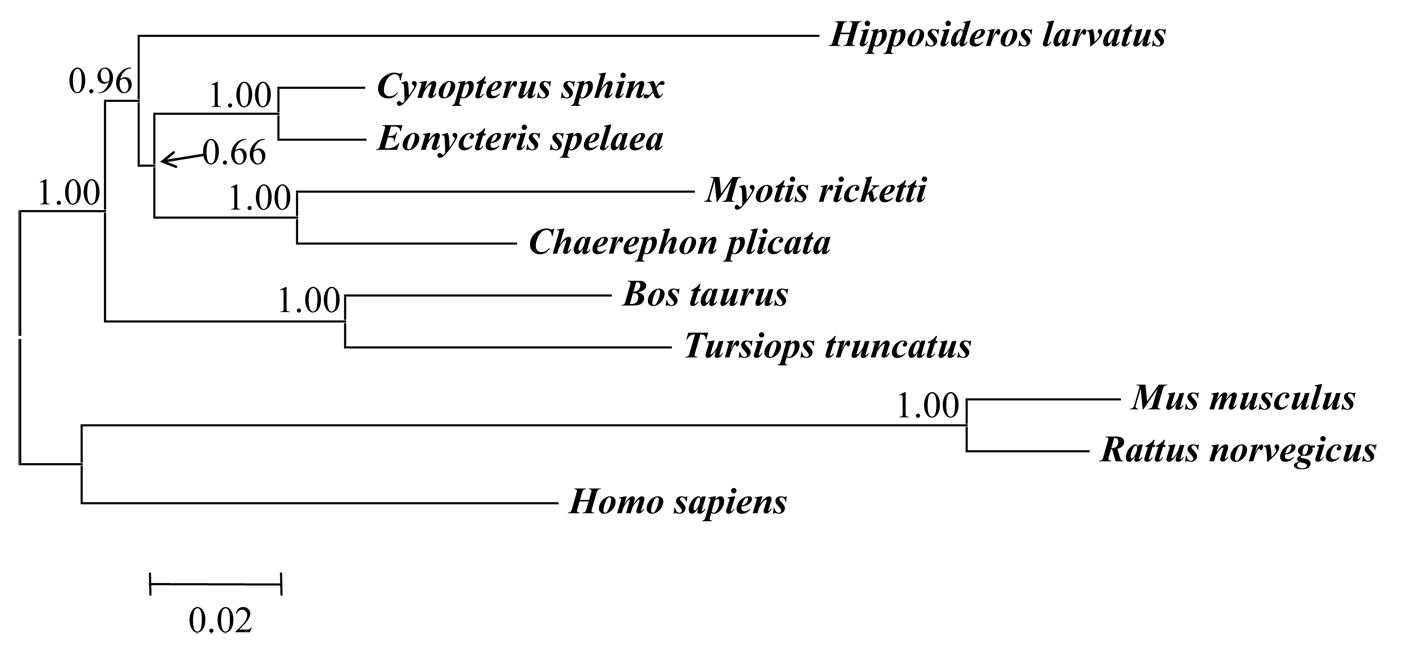

Supplement: Figure S5 — The BI tree for Pcdh15 based on the amino acid sequences excluding all parallel-evolved sites. (TIF) [file pgen.1002788.s005.tif]

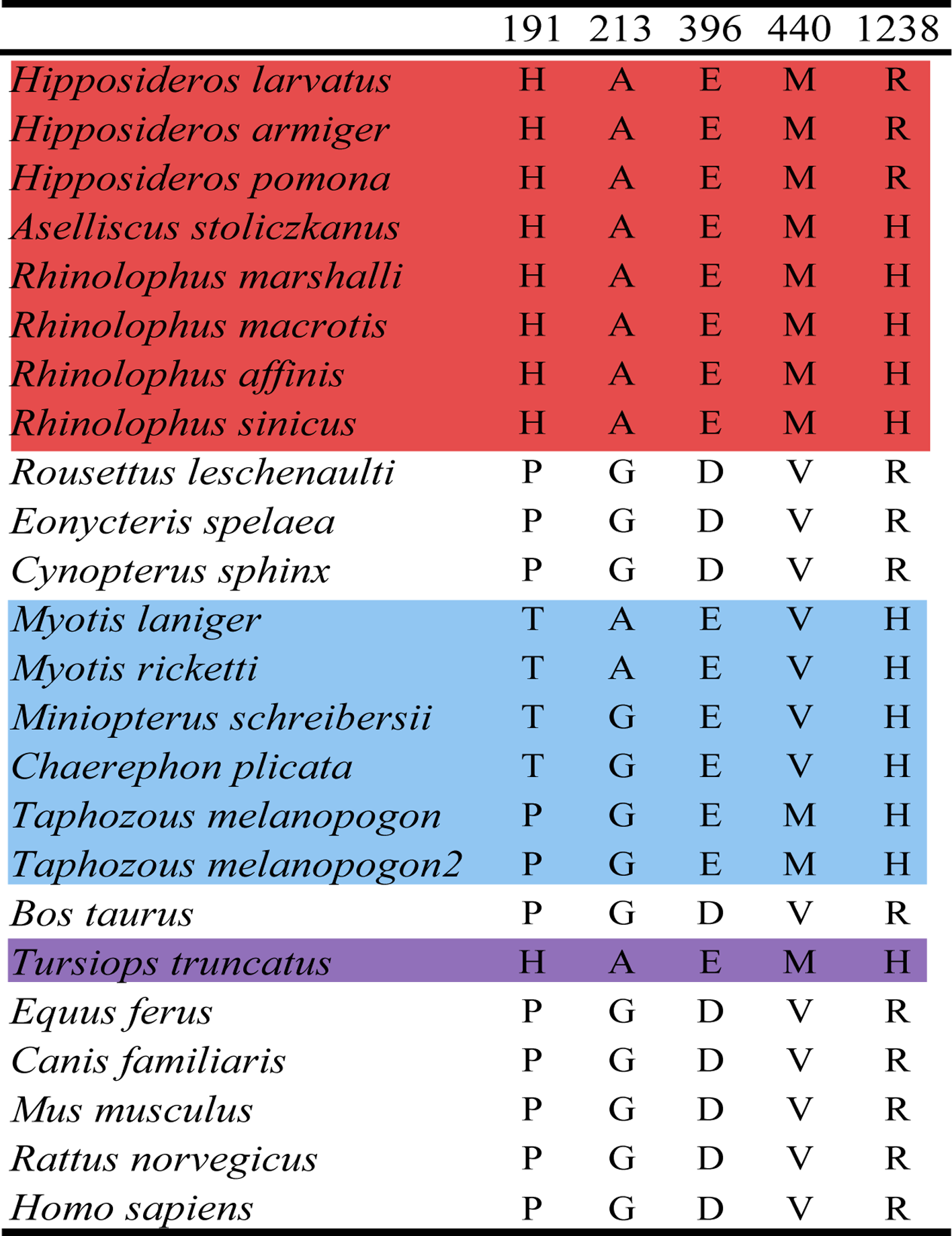

Supplement: Figure S6 — Parallel-evolved sites of Otof. (TIF) [file pgen.1002788.s006.tif]

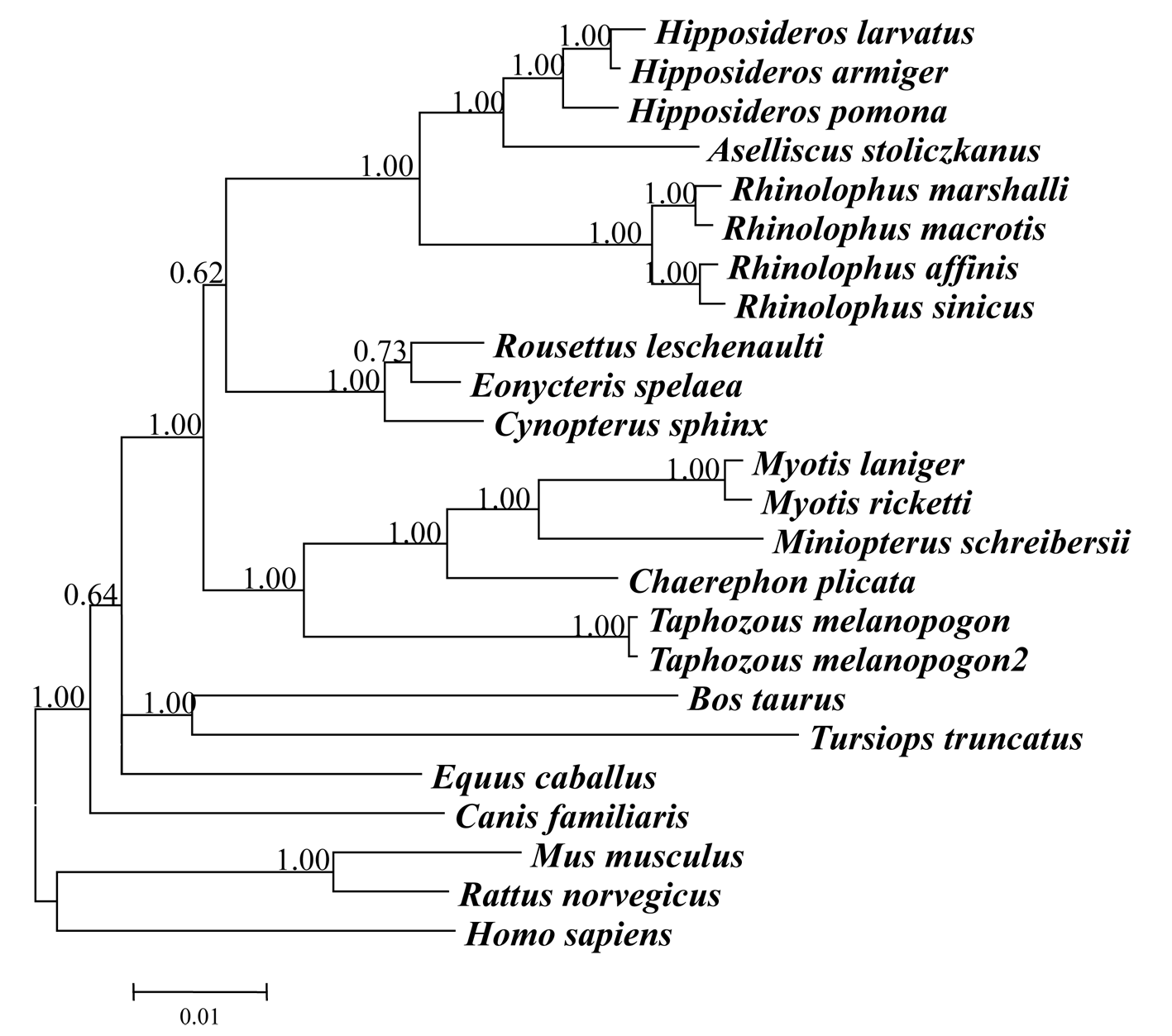

Supplement: Figure S7 — The BI tree for Otof based on the amino acid sequences excluding all parallel-evolved sites. (TIF) [file pgen.1002788.s007.tif]
