# Supplementary material for: Parallel Evolution of Auditory Genes for Echolocation in Bats and Toothed Whales
Source: PLoS Genet. 2012 Jun 28;8(6):e1002788. doi: 10.1371/journal.pgen.1002788 (PMC3386236; doi:10.1371/journal.pgen.1002788)
Supplement: Table S1 — Details of the selective pressure analyses on the three hearing genes. (DOCX) [file pgen.1002788.s008.docx]

1. **For *CDH23***

| **Model** | **Parameter Estimated** | **lnL** | **2⊿lnL**  **(*P* value)** | | **Positively Selected Sites (BEB Analysis)** |
| --- | --- | --- | --- | --- | --- |
| **One ratio** | ω = 0.0546 | -35796.7768 | 267.7826  **(*P* < 0.001)** | |  |
| **Free ratio** | ω_branch_ *_b_* = 0.1432  ω_branch_ *_d_* = 0.0290  ω_branch_ *_g_* = 0.1167 | -35662.8855 |  |  |  |
| **Branch Model** |  |  |  | |  |
| branch *b*  and  branch *d* | ω_branch (_*_b+d_*_)_ = 0.1107 ω_0_ = 0.0500 | -35771.7882 | 418.8219  **(*P* < 0.001)** | |  |
| branch *b*  and  branch *d*  ω_branch (_*_b+d_*_)_ = 1 | ω_0_ = 0.0483 | -35981.1991 |  |  |  |
| branch *g* | ω_branch_ *_g_* = 0.1145 ω_0_ = 0.0516 | -35779.9788 | 246.9333  **(*P* < 0.001)** | |  |
| branch *g*  ω_branch_ *_g_* = 1 | ω_0_ = 0.0505 | -35903.4455 |  |  |  |
| branch *b*, *d* and *g* | ω_echolocators_ = 0.1126 ω_0_ = 0.0465 | -35750.5605 | 660.6044  **(*P* < 0.001)** | |  |
| branch *b*, *d* and *g*  ω_echolocators_= 1 | ω_0_ = 0.0437 | -36080.8627 |  |  |  |
| **Branch-site Model** |  |  |  | |  |
| branch *b* | site class 0 1 2a 2b  proportion 0.92097 0.05178 0.02580 0.00145  background ω 0.03156 1.00000 0.03156 1.00000  foreground ω 0.03156 1.00000 3.85446 3.85446 | -35474.2382 | Test 1 | 71.4450  **(*P* < 0.001)** | **213 A 0.984***,  **692 F 0.984***, **1165 N 0.989***, **1171 S 0.958***, **1256 D 0.989***, **1356 I 0.990****, 1687 T 0.925, **2492 L 0.970*** |
|  |  |  | Test 2 | 10.4158  **(*P* < 0.010)** |  |
| branch *d* | site class 0 1 2a 2b  proportion 0.94313 0.05687 0.00000 0.00000  background ω 0.03347 1.00000 0.03347 1.00000  foreground ω 0.03347 1.00000 1.00000 1.00000 | -35509.9607 | Test 1 | 0  (*P* ≥ 0.995) |  |
|  |  |  | Test 2 | 0  (*P* ≥ 0.995) |  |
| branch *b*  and  branch *d* | site class 0 1 2a 2b  proportion 0.93530 0.05632 0.00790 0.00048  background ω 0.03338 1.00000 0.03338 1.00000  foreground ω 0.03338 1.00000 1.00000 1.00000 | -35509.8379 | Test 1 | 0.2455  (*P* <0.900) |  |
|  |  |  | Test 2 | 0  *P* ≥ 0.995 |  |
| branch *g* | site class 0 1 2a 2b  proportion 0.92922 0.05575 0.01418 0.00085  background ω 0.03168 1.00000 0.03168 1.00000  foreground ω 0.03168 1.00000 5.94936 5.94936 | -35482.5897 | Test 1 | 54.7421  **(*P* < 0.001)** | **580 S 0.974***,  **840 H 0.973***,  **1011 H 0.973***, **1014 T 0.978***, **2133 T 0.952***, **2224 T 0.974*** |
|  |  |  | Test 2 | 10.7224  **(*P* < 0.010)** |  |
| branch *b*, *d* and *g* | site class 0 1 2a 2b  proportion 0.92519 0.05501 0.01869 0.00111  background ω 0.03126 1.00000 0.03126 1.00000  foreground ω 0.03126 1.00000 3.17974 3.17974 | -35477.3803 | Test 1 | 65.1609  **(*P* < 0.001)** | **580 S 0.962***,  **840 H 0.961***, **1011 H 0.963***, **1014 T 0.970***, **1026 D 0.953***, **1233 E 0.960***, **1359 I 0.965***, 2042 T 0.939, 2133 T 0.936, **2224 T 0.964*** |
|  |  |  | Test 2 | 8.6179  **(*P* < 0.010)** |  |

1. **For *PCDH15***

| **Model** | **Parameter Estimated** | **lnL** | **2⊿lnL**  **(*P* value)** | | **Positively Selected Sites (BEB Analysis)** |
| --- | --- | --- | --- | --- | --- |
| **One ratio** | ω = 0.1231 | -15452.8512 | 206.3534  **(*P* < 0.001)** | |  |
| **Free ratio** | ω_branch_ *_b_* = 0.3530  ω_branch_ *_d_* = 0.2154  ω_branch_ *_g_* = 0.4474 | -15349.6745 |  |  |  |
| **Branch Model** |  |  |  | |  |
| branch *b*  and  branch *d* | ω_branch (_*_b+d_*_)_ = 0.3211 ω_0_ = 0.0998 | -15415.0079 | 83.0381  **(*P* < 0.001)** | |  |
| branch *b*  and  branch *d*  ω_branch (_*_b+d_*_)_ = 1 | ω_0_ = 0.0983 | -15456.5269 |  |  |  |
| branch *g* | ω_branch_ *_g_* = 0.4469 ω_0_ = 0.1109 | -15426.9934 | 16.6620  **(*P* < 0.001)** | |  |
| branch *g*  ω_branch_ *_g_* = 1 | ω_0_ = 0.1105 | -15435.3244 |  |  |  |
| branch *b*, *d* and *g* | ω_echolocators_ = 0.3561 ω_0_ = 0.0851 | -15380.3309 | 96.3532  **(*P* < 0.001)** | |  |
| branch *b*, *d* and *g*  ω_echolocators_= 1 | ω_0_ = 0.0834 | -15428.5075 |  |  |  |
| **Branch-site Model** |  |  |  | |  |
| branch *b* | site class 0 1 2a 2b  proportion 0.79672 0.11161 0.08040 0.01126  background ω 0.02983 1.00000 0.02983 1.00000  foreground ω 0.02983 1.00000 2.00038 2.00038 | -15181.4255 | Test 1 | 55.4773  **(*P* < 0.001)** | 430 R 0.934, **454 H 0.965***, **816 T 0.970*** |
|  |  |  | Test 2 | 2.6591  (*P* < 0.500) |  |
| branch *d* | site class 0 1 2a 2b  proportion 0.86720 0.12926 0.00308 0.00046  background ω 0.03721 1.00000 0.03721 1.00000  foreground ω 0.03721 1.00000 24.77802 24.77802 | -15204.2949 | Test 1 | 9.7385  **(*P* < 0.010)** | 573 T 0.921 |
|  |  |  | Test 2 | **6.4259**  **(*P* < 0.050)** |  |
| branch *b*  and  branch *d* | site class 0 1 2a 2b  proportion 0.86830 0.13170 0.00000 0.00000  background ω 0.03752 1.00000 0.03752 1.00000  foreground ω 0.03752 1.00000 1.00000 1.00000 | -15209.1642 | Test 1 | 0  (*P* ≥ 0.995) |  |
|  |  |  | Test 2 | 0  (*P* ≥ 0.995) |  |
| branch *g* | site class 0 1 2a 2b  proportion 0.81368 0.11630 0.06127 0.00876  background ω 0.03403 1.00000 0.03403 1.00000  foreground ω 0.03403 1.00000 2.72477 2.72477 | -15193.4511 | Test 1 | 31.4262  **(*P* < 0.001)** | **1584D0 .994**** |
|  |  |  | Test 2 | 2.9014  (*P* < 0.100) |  |
| branch *b*, *d* and *g* | site class 0 1 2a 2b  proportion 0.82047 0.11653 0.05516 0.00783  background ω 0.03394 1.00000 0.03394 1.00000  foreground ω 0.03394 1.00000 2.84765 2.84765 | -15193.1313 | Test 1 | 32.0658  **(*P* < 0.001)** | **1584D0.992****, 1936 L 0.947 |
|  |  |  | Test 2 | 3.5302  (*P* < 0.100) |  |

1. **For *OTOF***

| **Model** | **Parameter Estimated** | **lnL** | **2⊿lnL**  **(P value)** | | **Positively**  **Selected Sites (BEB Analysis)** |
| --- | --- | --- | --- | --- | --- |
| **One ratio** | ω = 0.0374 | -19845.3890 | 118.3583  **(P < 0.001)** | |  |
| **Free ratio** | ω_branch_ *_b_* = 0.0693  ω_branch_ *_d_* = 0.0356  ω_branch_ *_g_* = 0.0895 | -19786.2098 |  |  |  |
| **Branch Model** |  |  |  | |  |
| branch *b*  and  branch *d* | ω_branch (_*_b+d_*_)_ = 0.0572 ω_0_ = 0.0364 | -19843.5402 | 158.6241  **(P < 0.001)** | |  |
| branch *b*  and  branch *d*  ω_branch (_*_b+d_*_)_ = 1 | ω_0_ = 0.0350 | -19922.8522 |  |  |  |
| branch *g* | ω_branch_ *_g_* = 0.0886 ω_0_ = 0.0352 | -19835.1619 | 126.9486  **(P < 0.001)** | |  |
| branch *g*  ω_branch_ *_g_* = 1 | ω_0_ = 0.0341 | -19898.6363 |  |  |  |
| branch *b*, *d* and *g* | ω_echolocators_ = 0.0732 ω_0_ = 0.0340 | -19833.7703 | 278.1766  **(P < 0.001)** | |  |
| branch *b*, *d* and *g*  ω_echolocators_= 1 | ω_0_ = 0.0315 | -19972.8586 |  |  |  |
| **Branch-site Model** |  |  |  | |  |
| branch *b* | site class 0 1 2a 2b  proportion 0.95114 0.03918 0.00930 0.00038  background ω 0.02313 1.00000 0.02313 1.00000  foreground ω 0.02313 1.00000 3.10544 3.10544 | -19683.4542 | Test 1 | 5.9493  (P < 0.100) |  |
|  |  |  | Test 2 | 0.7696  (P < 0.500) |  |
| branch *d* | site class 0 1 2a 2b  proportion 0.95968 0.04032 0.00000 0.00000  background ω 0.02352 1.00000 0.02352 1.00000  foreground ω 0.02352 1.00000 1.00000 1.00000 | -19686.4288 | Test 1 | 0  (P ≥ 0.995) |  |
|  |  |  | Test 2 | 0  (P ≥ 0.995) |  |
| branch *b*  and  branch *d* | site class 0 1 2a 2b  proportion 0.95550 0.03924 0.00505 0.00021  background ω 0.02331 1.00000 0.02331 1.00000  foreground ω 0.02331 1.00000 2.34946 2.34946 | -19685.2083 | Test 1 | 2.4411  (P < 0.500) |  |
|  |  |  | Test 2 | 0.4690  (P < 0.500) |  |
| branch *g* | site class 0 1 2a 2b  proportion 0.92038 0.03758 0.04039 0.00165  background ω 0.02221 1.00000 0.02221 1.00000  foreground ω 0.02221 1.00000 1.00000 1.00000 | -19678.7638 | Test 1 | **15.3299**  **(P < 0.001)** | **944 L 0.950*** |
|  |  |  | Test 2 | 0  (P ≥ 0.995) |  |
| branch *b*, *d* and *g* | site class 0 1 2a 2b  proportion 0.93940 0.03477 0.02491 0.00092  background ω 0.02225 1.00000 0.02225 1.00000  foreground ω 0.02225 1.00000 1.36768 1.36768 | -19671.6820 | Test 1 | **29.4937**  **(P < 0.001)** | 191 P 0.925, 213 G 0.928, **396 D 0.989***, 944 L 0.931, 1238 R 0.916 |
|  |  |  | Test 2 | 0.4685  (P < 0.500) |  |
