# Supplementary material for: Parallel Evolution of Auditory Genes for Echolocation in Bats and Toothed Whales
Source: PLoS Genet. 2012 Jun 28;8(6):e1002788. doi: 10.1371/journal.pgen.1002788 (PMC3386236; doi:10.1371/journal.pgen.1002788)
Supplement: Table S2 — Expression levels of Otof in the Common Bent-wing Bat and Old World Fruit Bat. (DOCX) [file pgen.1002788.s009.docx]

| Species | Age | Region | Average Fold-change | Standard Deviation |
| --- | --- | --- | --- | --- |
| Common Bent-wing Bat (*Miniopterus schreibersii*) | Adult | Auditory cortex | 74.47 | 101.21 |
|  |  | Visual cortex | 40.74 | 18.75 |
|  |  | Motor and sensory cortex | 30.00 | 20.54 |
|  |  | Olfactory bulb | 16.84 | 18.84 |
|  |  | Cerebellum | 1.00 |  |
| Common Bent-wing Bat (*Miniopterus schreibersii*) | Embryo | Auditory cortex | 13.32 | 17.16 |
|  |  | Visual cortex | 3.79 | 2.99 |
|  |  | Motor and sensory cortex | 1.96 | 0.69 |
|  |  | Olfactory bulb | 1.82 | 1.64 |
|  |  | Cerebellum | 1.20 | 0.78 |
| Old-World Fruit Bat  (*Rousettus leschenaultia*) | Adult | Auditory cortex | 3.20 | 0.71 |
|  |  | Visual cortex | 3.54 | 0.27 |
|  |  | Motor and sensory cortex | 2.98 | 1.33 |
|  |  | Olfactory bulb | 0.33 | 0.15 |
|  |  | Cerebellum | 0.40 | 0.12 |
