# Supplementary material for: Parallel Evolution of Auditory Genes for Echolocation in Bats and Toothed Whales
Source: PLoS Genet. 2012 Jun 28;8(6):e1002788. doi: 10.1371/journal.pgen.1002788 (PMC3386236; doi:10.1371/journal.pgen.1002788)
Supplement: Table S3 — Species and their accession numbers for the genes Cdh23, Pcdh15, Otof, and Actb used in this research. (DOCX) [file pgen.1002788.s010.docx]

| Order | Family | Species | *Cdh23* gene | *Pcdh15* gene | *Otof* gene | *Actb* gene |
| --- | --- | --- | --- | --- | --- | --- |
| Primates | Hominidae | *Homo sapiens* | ENSG00000107736 | ENSG00000150275 | ENSG00000115155 | ENSG00000075624 |
| Rodentia | Muridae | *Mus musculus* | ENSMUSG00000012819 | ENSMUSG00000052613 | ENSMUSG00000062372 | ENSMUSG00000029580 |
| Rodentia | Muridae | *Rattus norvegicus* | ENSRNOG00000033087 | ENSRNOG00000000606 | ENSRNOG00000009967 | ENSRNOG00000034254 |
| Ruminantia | Bovidae | *Bos taurus* | ENSBTAG00000021497 | ENSBTAG00000045905 | ENSBTAG00000009650 | ENSBTAG00000026199 |
| Carnivora | Canidae | *Canis familiaris* | ENSCAFG00000014229 |  | ENSCAFG00000004385 | ENSCAFG00000016020 |
| Perissodactyla | Equidae | *Equus ferus* | ENSECAG00000023411 |  | ENSECAG00000002692 | ENSECAG00000015935 |
| Cetacea | Delphinidae | *Tursiops truncatus* | ENSTTRG00000011205 | ENSTTRG00000014014 | ENSTTRG00000010249 | ENSTTRG00000001953 |
| Chiroptera | Emballonuridae | *Taphozous melanopogon* | JF808088 |  | JQ284408, JQ284409 |  |
| Chiroptera | Hipposideridae | *Aselliscus stoliczkanus* | JF808082 |  | JQ284415 |  |
| Chiroptera | Hipposideridae | *Hipposideros armiger* | JF808087 | JF808091 | JQ284411 | JQ284420 |
| Chiroptera | Hipposideridae | *Hipposideros larvatus* | JF808083 |  | JQ284406 |  |
| Chiroptera | Hipposideridae | *Hipposideros pomona* |  |  | JQ284403 | JQ284427 |
| Chiroptera | Molossidae | *Chaerephon plicata* | JF808086 | JF808094 | JQ284416 | JQ284423 |
| Chiroptera | Rhinolophidae | *Rhinolophus affinis* |  |  | JQ284405 | JQ284430 |
| Chiroptera | Rhinolophidae | *Rhinolophus macrotis* |  |  | JQ284412 | JQ284419 |
| Chiroptera | Rhinolophidae | *Rhinolophus marshalli* |  |  | JQ284407 | JQ284422 |
| Chiroptera | Rhinolophidae | *Rhinolophus sinicus* |  |  | JQ284404 | JQ284429 |
| Chiroptera | Vespertilionidae | *Miniopterus schreibersii* | JF808081 |  | JQ284413 | JQ284418 |
| Chiroptera | Vespertilionidae | *Myotis laniger* | JF808085 | JF808092 | JQ284414 | JQ284425 |
| Chiroptera | Vespertilionidae | *Myotis ricketti* |  |  | JQ284410 | JQ284421 |
| Chiroptera | Pteropodidae | *Cynopterus sphinx* |  | JF808093 | JQ284402 |  |
| Chiroptera | Pteropodidae | *Eonycteris spelaea* | JF808084 | JF808090 | JQ284401 |  |
| Chiroptera | Pteropodidae | *Rousettus leschenaulti* | JF808089 |  | JQ284400 | JQ284428 |
