# Supplementary material for: Parallel Evolution of Auditory Genes for Echolocation in Bats and Toothed Whales
Source: PLoS Genet. 2012 Jun 28;8(6):e1002788. doi: 10.1371/journal.pgen.1002788 (PMC3386236; doi:10.1371/journal.pgen.1002788)
Supplement: Table S4 — The primers used for amplifying and sequencing the three hearing genes. (DOCX) [file pgen.1002788.s011.docx]

| **Gene Name** | **Species** | **Sample** | **Number of**  **Primers** | **Site** | **Primer sequences** |
| --- | --- | --- | --- | --- | --- |
| *Cdh23* | Chiroptera | cDNA | 1 | 5 | F5’-3’ GGTGCCACGTYRCCACCAGC |
|  |  |  |  | 1247 | R5’-3’ GCCACYCGGATGCGAATGTC |
|  |  |  | 2 | 714 | F5’-3’ CAACCTGCCYTACAGCACCAA |
|  |  |  |  | 1929 | R5’-3’ GGCCATGACYGTCAGATAAA |
|  |  |  | 3 | 1841 | F5’-3’ TGTATGARGGCTATGGAGTGAT |
|  |  |  |  | 3020 | R5’-3’ TCGGACACGGAGACRTTGTA |
|  |  |  | 4 | 2617 | F5’-3’ AGCAGTGCCACRGTGTTTGT |
|  |  |  |  | 3853 | R5’-3’ CRTTCATCAGGTAGCTGGTCTT |
|  |  |  | 5 | 3733 | F5’-3’ CTGGTRAACTACCGCATCCT |
|  |  |  |  | 5027 | R5’-3’ GCRTAGGTMACTGTGCCATT |
|  |  |  | 6 | 4526 | F5’-3’ CACGGAAGAAGRACCACATC |
|  |  |  |  | 6008 | R5’-3’ TAGGTCACCACRGCATAGAC |
|  |  |  | 7 | 5705 | F5’-3’ TCKCCATCAATGCCACGACA |
|  |  |  |  | 7188 | R5’-3’ CACAATCTCCAGGTAGACRGG |
|  |  |  | 8 | 6694 | F5’-3’ GAYGCCTTTGCYGTGAATAT |
|  |  |  |  | 7925 | R5’-3’ TCTGTRGCRTAGACCTCGTA |
|  |  |  | 9 | 7897 | F5’-3’ TCCAAYGTGTACGAGGTCTA |
|  |  |  |  | 9036 | R5’-3’ CAGCTCTGTYTGTGCGAAGT |
|  |  |  | 10 | 8398 | F5’-3’ GCYGTCTTCTCCTTCATYGT |
|  |  |  |  | 9687 | R5’-3’ TTTGAGCCGCAGGTAATCCT |
|  |  |  | 11 | 8997 | F5’-3’ GGAYAAGAAGGGTCGGGTGA |
|  |  |  |  | 10013 | R5’-3’ GTKGATTTGGCGGACTCYGT |
|  | Cetacea | DNA | 1 | 980 | F5’-3’ GCATCCTCTATTCCATCCTTGTT |
|  |  |  |  | 1100 | R5’-3’ TTCAGCCTTAATAACCAAATCAAAT |
|  |  |  | 2 | 2000 | F5’-3’ CGAGAGGGAGACCCAATAACAT |
|  |  |  |  | 2080 | R5’-3’ TTTCCCTAAGGTTAGAATCCCAG |
|  |  |  | 3 | 2550 | F5’-3’ CGTTGAAGAGAATCTGCCAGC |
|  |  |  |  | 3040 | R5’-3’ GGTTCTTCATTAAGATTGACTCGAG |
|  |  |  | 4 | 3720 | F5’-3’ CGCATTCTGGAGATCCGG |
|  |  |  |  | 3860 | R5’-3’ ACTTTAAACTGTCTGTAGCTGACCAA |
|  |  |  | 5 | 4240 | F5’-3’ ATTGATATCTGATGAGGATGCCTT |
|  |  |  |  | 4280 | R5’-3’ AATCTGTTCTTwGTGAAAAGTCTGAATTT |
|  |  |  | 6 | 4040 | F5’-3’ TGGGGACAGCACAATGCATA |
|  |  |  |  | 4610 | R5’-3’ TAAGGCATCCTCATCAGATATCAAT |
| *Pcdh15* | Chiroptera | cDNA | 1 | 238 | F5’-3’ AACGWGGAYTACTGGGWGC |
|  |  |  |  | 1425 | R5’-3’ KTSCTGTTCYTCCCTGTCCAC |
|  |  |  | 2 | 1235 | F5’-3’ CAACTATTTCCGACAGCGT |
|  |  |  |  | 2249 | R5’-3’ CCATTTATTCCAGCATCAGG |
|  |  |  | 3 | 2012 | F5’-3’ CCTTAGGGAAAGCMCTGGA |
|  |  |  |  | 3111 | R5’-3’ TTGAGAGAAGCACGGGAT |
|  |  |  | 4 | 3033 | F5’-3’ TGGTGAGNNNGTGATGTCAG |
|  |  |  |  | 4103 | R5’-3’ TCTCCTCGCTTCTTGATGC |
|  |  |  | 5 | 4018 | F5’-3’ GACTTTCAACCGCATTATGG |
|  |  |  |  | 4966 | R5’-3’ CCAACTGGTCTGTTCCTTGTA |
|  |  |  | 6 | 4770 | F5’-3’ GCCCAATAGACTGGATACGA |
|  |  |  |  | 5522 | R5’-3’ GAAGTTGAAGGAGGKAGTGG |
|  | Cetacea | DNA | 1 | 661 | F5’-3’ CAGCAGCAGTACAGCCGC |
|  |  |  |  | 1949 | R5’-3’ TAGTGRGGCTKCTGGAAC |
|  |  |  | 2 | 1756 | F5’-3’ ACNTTCCGCATGGACCGC |
|  |  |  |  | 2648 | R5’-3’ TGGGCGACGAAGCTGGAG |
|  |  |  | 3 | 2576 | F5’-3’ ACCCTGTGCTGCTGAACC |
|  |  |  |  | 3647 | R5’-3’ GGRTTGAGGTCACGGTCG |
|  |  |  | 4 | 3555 | F5’-3’ CCTCAACCCCATCCAGAC |
|  |  |  |  | 4398 | R5’-3’ CCGTNGTGGGGTTGATGG |

| *Otof* | Chiroptera | cDNA | 1 | 52 | F5’-3’ ARGGGSGACCGGATCGCCAAAG |
| --- | --- | --- | --- | --- | --- |
|  |  |  |  | 789 | R5’-3’ CRATCACTGTGATGCTGACCTG |
|  |  |  | 2 | 285 | F5’-3’ GGTGACYGACACGCTGATTG |
|  |  |  |  | 1380 | R5’-3’ AAAGAAGACTTGCACRTAGGG |
|  |  |  | 3 | 1300 | F5’-3’ ACMAGCCTCATGGCCAACGTG |
|  |  |  |  | 2277 | R5’-3’ CTCGGGGTAGGACTTCTCCGT |
|  |  |  | 4 | 2073 | F5’-3’ ACCCATGCGGCCCCAGATCACT |
|  |  |  |  | 2911 | R5’-3’ GCTGCGRGCCTGGTACATGTG |
|  |  |  | 5 | 2761 | F5’-3’ AGCAAGCARCGCAAGGA |
|  |  |  |  | 3591 | R5’-3’ GTTCTCHGGGAGGTCCAC |
|  |  |  | 6 | 3544 | F5’-3’ AACTTCARCACCCTSGTCAAG |
|  |  |  |  | 4256 | R5’-3’ AGCTCCTTGGGGTACACCTT |
|  |  |  | 7 | 4074 | F5’-3’ GGAGAAGGAGGAGAYRGACAACA |
|  |  |  |  | 4937 | R5’-3’ AYGCGGTTGGACACCTTCACTCT |
|  |  |  | 8 | 4550 | F5’-3’ CTGACCCCTACATCGCCATCC |
|  |  |  |  | 5387 | R5’-3’ CGCCAGTTGAAGTTGCCCTC |
|  |  |  | 9 | 5411 | F5’-3’ TGGCTGCCGAGGARAAGAT |
|  |  |  |  | 5823 | R5’-3’ GGGTTTCTCYAGGGRATCAGGT |
|  | Cetacea | DNA | 1 | 76931 | F5’-3’ CTGCCTGCCCGCAGCTGATGCC |
|  |  |  |  | 77646 | R5’-3’ GGGGGGGTCCTGGGTCTTCTCC |
|  |  |  | 2 | 82672 | F5’-3’ AAGGGGCTGATGAAGGGCAA |
|  |  |  |  | 83339 | R5’-3’ GCAACTCTGAGGGGGGTGGA |
| *Actb* | Chiroptera | cDNA | 1 | 226 | F5’-3’ GTCACCAACTGGGACGACA |
|  |  |  |  | 1057 | R5’-3’ GGAAGGTGGACAGYGAGGC |
