# Supplementary material for: Parallel Evolution of Auditory Genes for Echolocation in Bats and Toothed Whales
Source: PLoS Genet. 2012 Jun 28;8(6):e1002788. doi: 10.1371/journal.pgen.1002788 (PMC3386236; doi:10.1371/journal.pgen.1002788)
Supplement: Table S5 — Details of the TaqMan gene expression assays. (DOCX) [file pgen.1002788.s012.docx]

| **Gene Name** | **Assay Name** | **Assay ID** | **Direction** | **Sequences** | **Base** | **Amplicon Size** | **Reporter Sequences** | **Base** | **Reporter**  **/Quencher** |
| --- | --- | --- | --- | --- | --- | --- | --- | --- | --- |
| *Otof* | GYYXY | AI1RUT6 | Forward | AGCCTCTGTCACAGCTCTCA | 20 | 108 | CCAATGTCTCCAACAAGC | 18 | FAM/MGB |
|  |  |  | Reverse | GTGATGCTGACCTGGTAATCCAT | 23 |  |  |  |  |
| *Actb* | GYYXYNC | AI20S0E | Forward | CCTCCCCCACGCCAT | 15 | 73 | CCTGCGTCTGGACCTG | 16 | FAM/MGB |
|  |  |  | Reverse | CGTGAGGATCTTCATGAGGTAGTC | 24 |  |  |  |  |
